# Supplementary material for: Estimation of Individual Positive Anti-Islet Autoantibodies from 3 Screen ICA Titer
Source: Int J Mol Sci. 2024 Jul 11;25(14):7618. doi: 10.3390/ijms25147618 (PMC11277171; doi:10.3390/ijms25147618)
Supplement: Supplementary file 1 [file ijms-25-07618-s001.zip › FigureS1.pptx]

## Slide 1
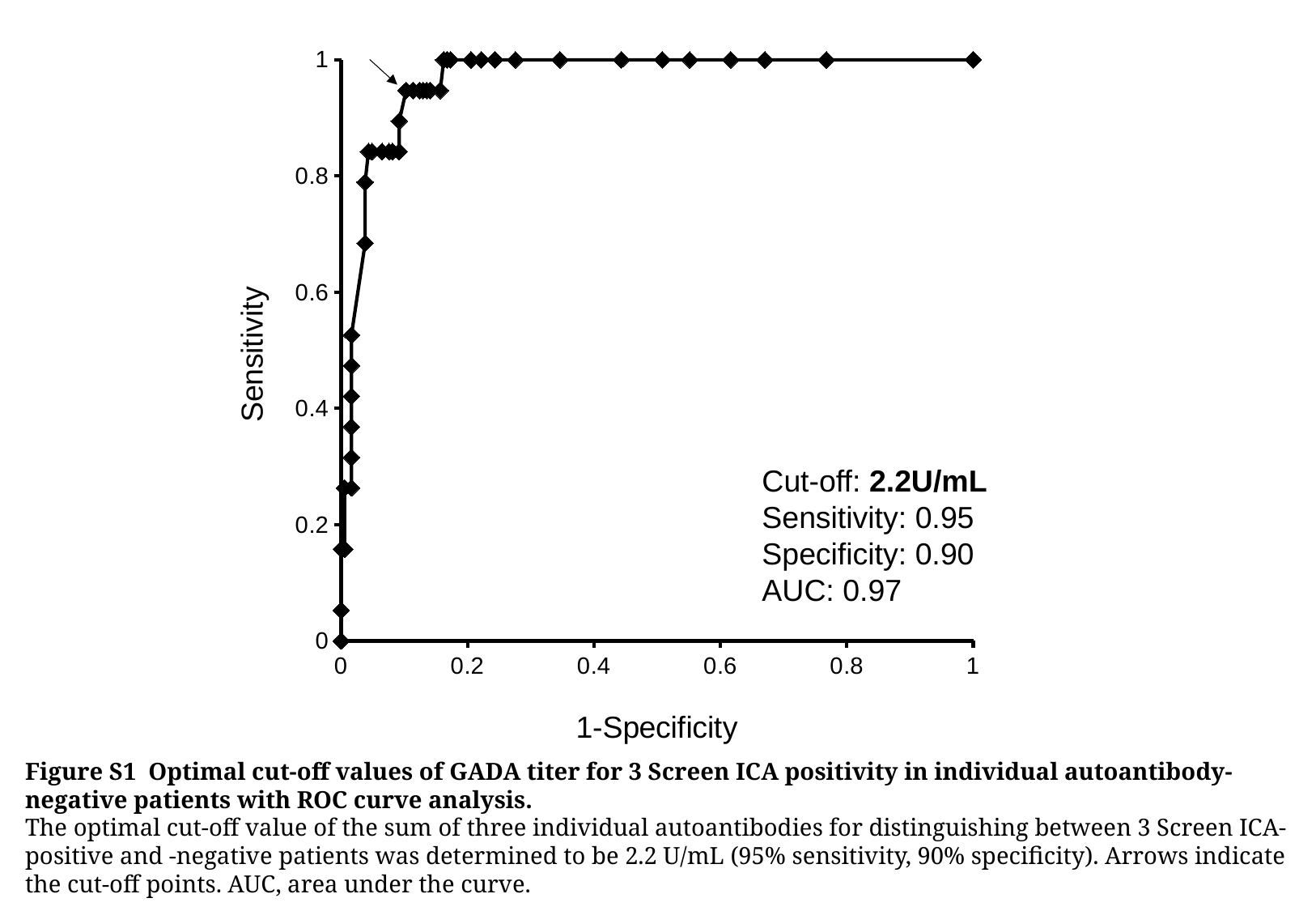

### Chart
| Category | GADA |
|---|---|Cut-off: 2.2U/mL
Sensitivity: 0.95
Specificity: 0.90
AUC: 0.97
Figure S1 Optimal cut-off values of GADA titer for 3 Screen ICA positivity in individual autoantibody-negative patients with ROC curve analysis.
The optimal cut-off value of the sum of three individual autoantibodies for distinguishing between 3 Screen ICA-positive and -negative patients was determined to be 2.2 U/mL (95% sensitivity, 90% specificity). Arrows indicate the cut-off points. AUC, area under the curve.
