# Supplementary material for: Estimation of Individual Positive Anti-Islet Autoantibodies from 3 Screen ICA Titer
Source: Int J Mol Sci. 2024 Jul 11;25(14):7618. doi: 10.3390/ijms25147618 (PMC11277171; doi:10.3390/ijms25147618)
Supplement: Supplementary file 1 [file ijms-25-07618-s001.zip › TableS1.pdf]

Table S1 Clinical characteristics

	Total (n=617)	Acute-onset type 1 diabetes (n=436)	SPIDDM (n=181)
Female (%)	307 (49.8)	215 (49.3)	92 (50.8)
Age at diagnosis (years)*	34 (0-83)	26 (0-74)	48 (8-83)
Duration (years)*	16 (0-66)	17 (0-66)	12 (0-45)
BMI (kg/m^2)**	23.4±3.8	22.9±3.6	24.4±3.9
Insulin dose (U/day)**	38.9±20.0	41.2±19.5	21.5±18.7
HbA1c (%)**	7.8±1.2	7.9±1.2	7.5±1.1
C-peptide (ng/mL)**, #	0.7±1.2	0.3±0.5	1.6±1.7
Autoimmune disease (%)##	98/612 (16.0)	65/433 (15.0)	33/179 (18.4)
3 Screen ICA positive (%)	424 (68.7)	294 (67.4)	130 (71.8)

Data are n (%), median (range), or Mean±SD

*Median (range), **Mean±SD

#C-peptide data were available in 266 patients with acute-onset type 1 diabetes and 118 SPIDDM.

##Data on the autoimmune diseases were not available in 5 patients.

SPIDDM, slowly-progressive type 1 diabetes
